# Pigeon as a model to study peripheral projections from the horizontal semicircular canal vestibular apparatus to a brainstem target immunoreactive for AMPA

**DOI:** 10.1590/ACB361206

**Published:** 2022-01-05

**Authors:** João Roberto Rocha, Maria de Fátima Passetto, Julianne dos Santos Maldonado-Menetti, Ana Lúcia Beirão Cabral, Claudio Antônio Barbosa de Toledo, Marcia Koike

**Affiliations:** 1 MSc. Postgraduate Program in Health Science - Instituto de Assistência Médica ao Servidor Público Estadual de São Paulo (IAMSPE) – Sao Paulo (SP), Brazil.; 2 MSc. Neuroscience Laboratory. Universidade Cidade de São Paulo (UNICID) – Sao Paulo (SP), Brazil.; 3 MSc. Fellow PhD degree. Postgraduate Program in Health Science - Instituto de Assistência Médica ao Servidor Público Estadual de São Paulo (IAMSPE) – Sao Paulo (SP), Brazil.; 4 PhD. Neuroscience Laboratory - Universidade Cidade de São Paulo (UNICID) – Sao Paulo (SP), Brazil.; 5 PhD. Neuroscience Laboratory - Universidade Cidade de São Paulo (UNICID), and Postgraduate Program in Health Science - Instituto de Assistência Médica ao Servidor Público Estadual de São Paulo (IAMSPE) – Sao Paulo (SP), Brazil.; 6 PhD. Postgraduate Program in Health Science - Instituto de Assistência Médica ao Servidor Público Estadual de São Paulo (IAMSPE), and Emergency Medicine Laboratory - Universidade de São Paulo (USP) - Sao Paulo (SP), Brazil.

**Keywords:** Mesenchymal Stem Cells, Endothelium, Blood Vessels, Peripheral Arterial Disease

## Abstract

**Purpose:**

To evaluate whether the pigeon (*Columba livia*) is a good model for evaluating the vestibular system involved with postural maintenance during movement.

**Methods:**

This study maps the brainstem targets of the horizontal ampullary inputs from the vestibular periphery of the pigeon. We used biotin dextran amine (BDA) injection in horizontal semicircular canal (HSCC), immunohistochemistry for GluR2/3 and GluR4 AMPA and computerized histomorphology reconstruction.

**Results:**

Our results show the same distribution pattern with ipsilateral projections to vestibular nuclear complex (VNC) from the HSCC, with the majority of labeled fibers being, long, thin, with few varicosities and many ramifications. Horizontal semicircular canal projections achieve neurons belonging to all nuclei of the VNC with exception of dorsal portion of lateral vestibular nucleus and this area express GluR2/3 and GluR4 AMPA receptors reinforcing the idea of glutamate participation in these connections.

**Conclusions:**

Pigeon is an appropriated experimental model to study of projections of HSCC and reinforcing the information that the vestibular system has strong relation with the fast responses necessary for postural control. Moreover, its phylogenetic organization apparently conservation, also seems to be a fundamental characteristic for vertebrates.

## Introduction

Maintaining the equilibrium and to provide postural adjustment in face with the gravity force is a fundamental requirement of all vertebrate species. Distinct animal classes utilize diverse locomotor strategies, but all of these properties are dependent of peripheral information offered by the vestibular organs and the existence of brainstem nuclei that receive and process such data, in order to provide descendent outputs to somatic motor neurons^1^^-^ 8.

Briefly speaking, the vestibular inputs of vertebrates come from the ampullae’s and macula’s hair cells present in the cupula of the semicircular canals (ampullary crista) and in the interior of the sacculus and the utricle (saccular macula and utricular macula), respectively^4^^,^ 9 ^,^ 10. Those hair cells are innervated by glutamic acid vestibular bipolar neurons in which perikarya are in the vestibular nerve ganglion (formally the Scarpa’s ganglion). The output branch (central portion) of these neurons composes the vestibular nerve that joins the cochlear nerve to constitute the eighth cranial nerve carrying vestibular signals to the vestibular nuclear complex (VNC)^1^^,^ 4 ^,^ 7 ^,^ 11 ^-^ 13.

**Figure 1:**
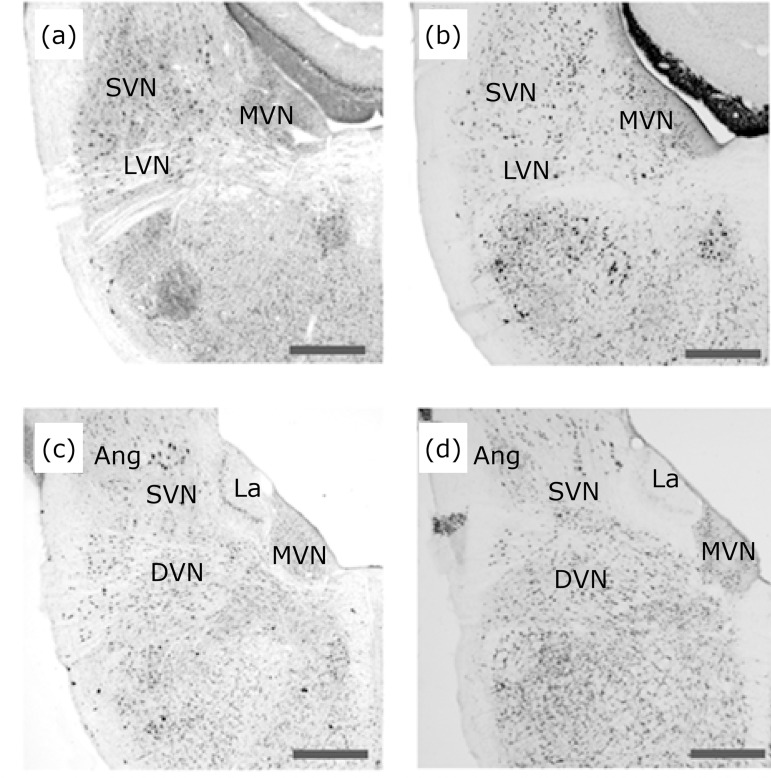
Location of VNC nuclei in cross-sections of the pigeon’s brainstem. Images **(a)** and **(c)** are the rostral and caudal locations, respectively, of the superior vestibular nucleus (SVN), lateral vestibular nucleus (LVN), medial vestibular nucleus (MVN) and descending vestibular nucleus (DVN) stained with Giemsa. Images **(b)** (rostral) and **(d)** (caudal) illustrate VNC neurons immunolabeled with NeuN reinforcing the location of the vestibular nuclei stained by Giemsa. In images **(c)** and **(d)**, it is also possible to observe the Angular (Ang) and laminar (La) nuclei. Scale bar: 1 mm.

The VNC is composed of the superior vestibular nucleus (SVN), lateral vestibular nucleus, medial vestibular nucleus and the descendent vestibular nucleus; although some additional small neuronal populations also participate^1^^,^ 2 ^,^ 4 ^,^ 14 ^-^ 16 (Fig. 1). The VNC neurons are rich in AMPA-type glutamic acid receptors^16^^-^ 18, supposedly targeted by the glutamic acid of Scarpa’s ganglion neurons, but the characteristics of these connections are missing. While many pharmacological properties are familiar^12^^,^ 13 ^,^ 18 ^-^ 20 the morphological details of its circuitry remain unknown.

Some species, such as primates, sustain complex motor actions with the horizontal plane movement prevailing. An avian is also able to keep their head in parallel stance to the surface during their walking and flying, suggesting that the horizontal semicircular canal (HSCC) is the preponderant source of information, similar for mammalian species^4^^,^ 21. To identify the central targets of vestibular system a biotin dextran amine (BDA) was injected as a neuronal tracer into the HSCC of pigeons and the immunohistochemical technique was also used to recognize the presence of subunits of the AMPA-type glutamate receptors (AMPArs) in the VNC area. Our group had previously demonstrated the distinction of the AMPArs in this area^16^, raising the hypothesis that the GluR2/3 and GluR4 subunits could play a key role in this entry system. Thus, the current purpose is to map the main brainstem targets of the horizontal ampullary inputs, from the vestibular periphery of the pigeon, together with the characterization of the AMPA-positive recipient neurons that receive its information.

## Methods

All experiments were conducted according to an approved institutional animal experimentation protocol (Committee of Ethical Research, process number 13259324), according to the Guide for the Care and Use of Laboratory Animals in Research of the National Institutes of Health, USA.

Fifteen adult (12 to 24 months) pigeons (*Columba livia*) weighing 360 to 420 g with no sex distinction were maintained in an animal facility room at 21°C with a 12 h-12 h dark-light cycle with food and water *ad libitum*.

All the animals were anesthetized by intramuscular injections of xylazine (1 mg/100 g of body weight) and ketamine (5 mg/100 g). The bony labyrinth was exposed and the horizontal canal was reached by an upper aperture to avoid damage to the auricular space that was used to fix the pigeon at the stereotaxic. A final perforation was completed with an insertion of a glass borosilicate micropipette tip (15 to 25 µm) used to inject the tracer solution, constituted by about 0.2 to 0.3 µL of a 10% solution of BDA of 10,000 molecular weight (BDA 10 kDa) dissolved in 0.1 mol L^–1^ of phosphate buffer (PB), pH 7.4^22^.

After 7–10 days the pigeons were deeply anesthetized as above, perfused and the brains were then removed, post-fixed and immersed in a cryoprotective solution^16^. Coronal frozen sections (40 µm) were cut with a cryostat (Leica, model CM 3050S) and collected into six separate compartments.

The histochemical detection with BDA label was performed with eleven animals distributed in five compartments, visualized using 0.05% diamino benzidine (DAB, Sigma Co. Saint Louis, MO, USA) and 0.03% hydrogen peroxide in PB with the addition of 1% nickel sulfate^22^. The last compartment was used for histochemical detection of the BDA together with immunodetection of the NeuN (1:1000; MAB377, Chemicon, Temecula, CA, USA), a protein that is largely found in neurons^23^, in order to facilitate the recognition of the VNC neuronal population.

With the last four pigeons, a double labeling was done between the BDA and glutamate receptors of the AMPA type: GluR1 (Chemicon, Temecula, CA, USA), GluR4 (Abcam Inc., Cambridge, MA, USA) or a single antibody to identify the presence of GluR2 and/or GluR3 (Chemicon, Temecula, CA, USA or Abcam Inc., Cambridge, MA, USA). The antibodies were diluted 1:500 in PB, incubated in biotin secondary antiserum, processed with the ABC-Elite kit (Vector Labs) and finally revealed by DAB^16^. Sections were rinsed in PB mounted on subbed slides for a minimum of two days before dehydrated and coverslipped with Permount (Fisher, Sci. Co., Pittsburg, PA, USA).

The images were captured by selecting brain regions with a 10× ocular of a Nikon Eclipse E 800 (Nikon Corporation, Chiyoda-Ku, Tokyo-To, Japan), a microscope equipped with a CCD camera (Optronics MagnaFire, Goleta, CA, USA) connected to a Macintosh desktop computer, and figures were prepared using the Adobe Photoshop software. No attempt was made to quantify the labeled structures or to evaluate the intensity of the markers, but when needed, we estimated the cellular size by measuring along the larger neuronal axis as priory described^16^.

Some general controls for the specificity of the AMPA immuno-staining were previously reported^24^^-^ 27, but to validate our own results incubation without the primary antibodies was performed. For western blotting assay 30 µg of total protein cell extract from pigeon hindbrain were separated onto an SDS-PAGE, transferred to a nitrocellulose membrane and incubated with GluR1, GluR2/3, GluR4 or preimmune serum with a 1:500 dilution in TBST. Revelation was done using a 1:1,000 antirabbit peroxidase conjugated antibody and the ECL (GE Healthcare) detection kit.

## Results

The obtained results were very similar for all animals assayed (the case number 2 was selected as a model) in which BDA was injected into the HSCC of pigeons. As shown in Fig. 1, all nuclei and their subdivisions compounding the pigeon VNC^4^^,^ 16 receive ipsilateral primary inputs from the HSCC by the ampullary nerve, except for the dorsal portion of the lateral vestibular nucleus.

### Vestibular nuclear complex afferents from the HSCC

**Figure 2:**
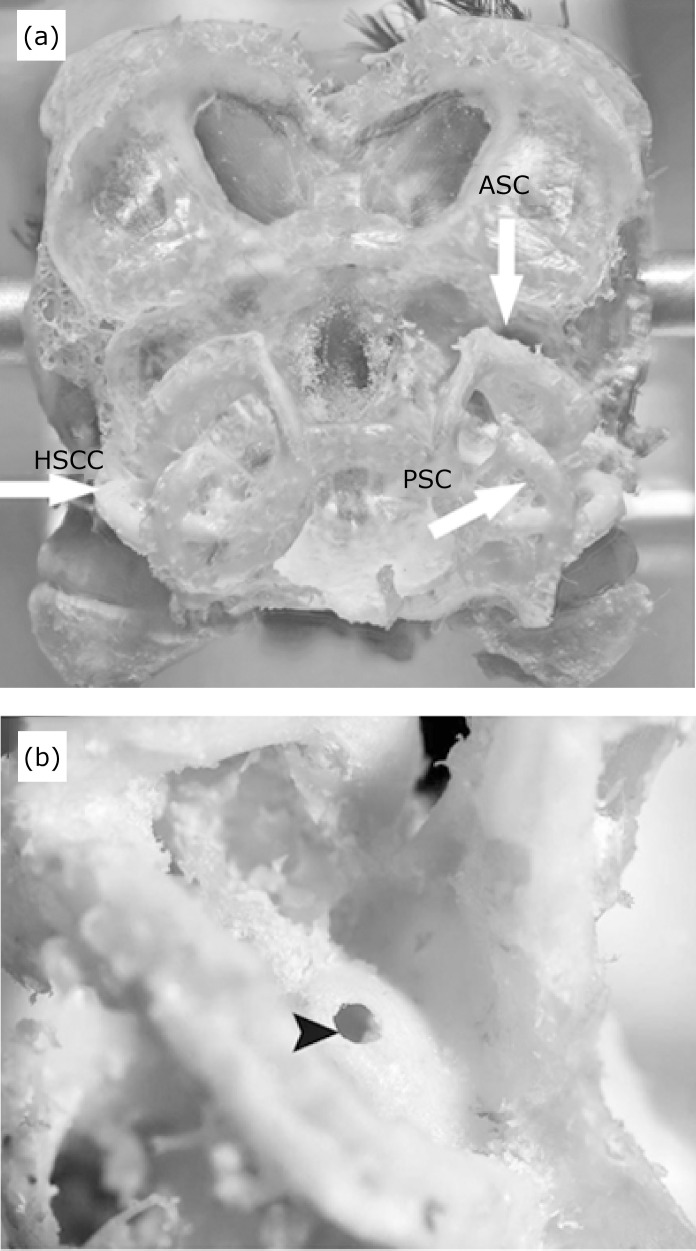
Spatial organization of semicircular canals after temporal bone and occipital lobe dissection. **(a)** White arrows show the horizontal semicircular canal (HSCC), anterior semicircular canal (ASC) and posterior semicircular canal (PSC). **(b)** Arrowhead indicates the injection area of biotinylated amine dextran (BDA) tracer into the HSC.

The vestibular ganglion (VG) of the injected side offered an unequivocal presence of neurons filled with BDA, almost dorsal, with virtually none positioned ventrally (Fig. 2a). Also visible, these BDA-containing neurons at dorsal VG constitute two populations, medium to large-sized (between 20 to 45 µm) and giant neurons (over 50 µm). Observing the arriving BDA-containing fibers, results showed that they occur at three distinct shapes: 1) long, thin and nonvaricose fibers with lateral ramifications, running to all directions over the VNC; 2) long, thin with many varicosities seeming to terminate at the nucleus area; and 3) large, short fibers holding intense labeling (Fig. 2b). The second type constantly presents a basket-form suggesting terminal fields also seeming to surround unlabeled cellular bodies (Fig. 2b, gray arrowhead).

**Figure 3:**
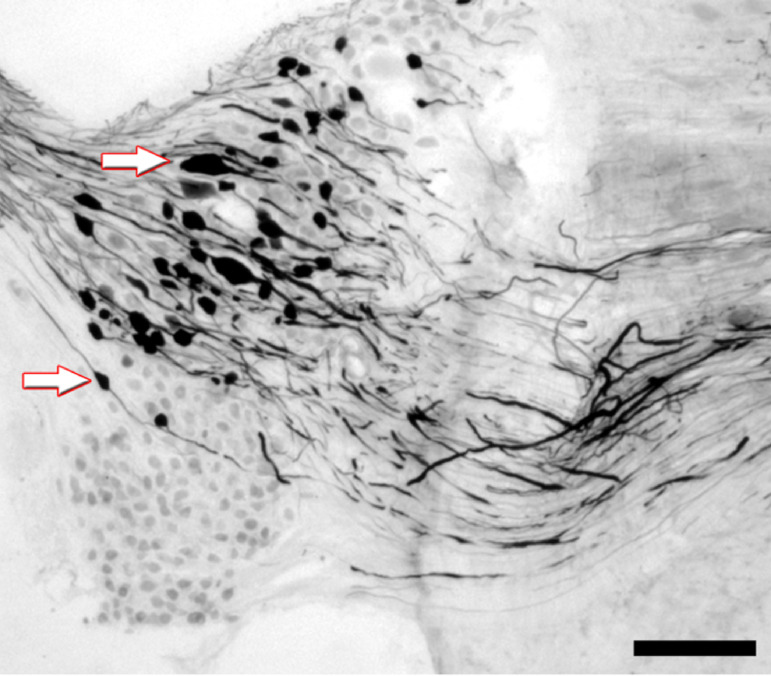
PDA labeling pattern in VG neurons. Arrows indicate bipolar neurons of different sizes, preferably located in the dorsal portion of the VG. Fibers from these neurons occupy the ventral position in the brainstem. Scale bar: 30 µm.

None BDA-labeled fibers were found contralateral to the injection side (Fig. 3). In addition, none BDA fibers were found in angular, laminar, or magnocellular nuclei but they were detected a reaching all nuclei of the ipsilateral VNC excluding, as cited, the dorsal part of the lateral vestibular nucleus and the appearance of these basket-like arrangements strongly suggest a reaching target, however, the occurrence over each nucleus differs in location as it follows (Fig. 3).

### Superior Vestibular Nucleus (SVN)

**Figure 4:**
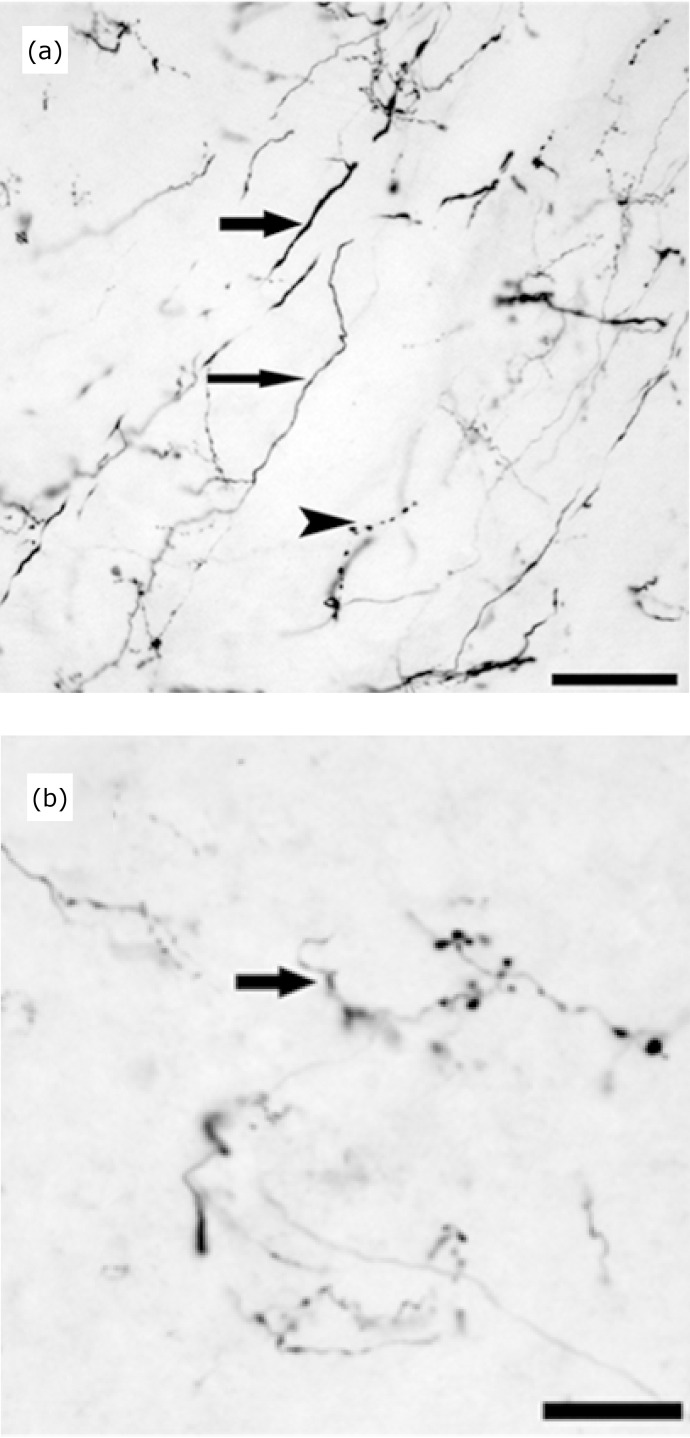
Types of fibers projecting to the VNC. Image **(a)** illustrates three types of fibers marked with PDA: long fiber, fine and nonvaricose (*thin arrow*); fine and varicose fiber (*arrowhead*), short and caliber fiber (*wide arrow*). Possible presence of a basket-shaped terminal button that surrounds a probable cell body observed in image **(b)** (*arrow*). Scale bars: A: 300 µm; B: 30 µm.

Concerning typology, the majority of the BDA-labeling was found to be in long and thin fibers containing few varicose and many ramifications, preferentially in the rostral SVN (Fig. 4a). These fibers were more evident in the dorsal-middle area of the rostral pole, where many passing fibers were also detected ascending to the cerebellar nuclei (Fig. 4a) and crossing in a medial direction to the medial vestibular nucleus (Fig. 4b). In the central SVN area these thin varicose fibers form branches resembling terminal fields that seem to embrace blank structures, possibly unlabeled neurons (gray arrow head in Fig. 2b).

### Lateral Vestibular Nucleus (LVN)

**Figure 5:**
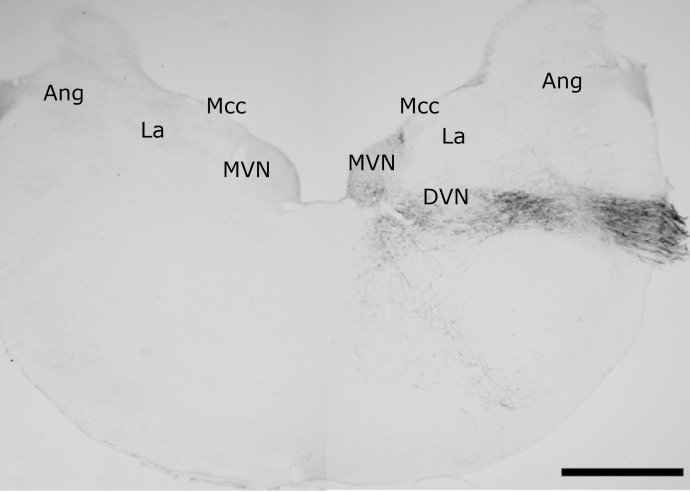
Projection of fibers from the horizontal semicircular canal (HSCC) to the VNC. The image shows the entrance of PDA-filled fibers into the nuclei of the caudal vestibular complex (VNC) ipsilateral to the side of the injection and the absence of projections on the contralateral side. Note the presence of fibers in the descending vestibular nucleus (DVN) and medial vestibular nucleus (MVN) and absence of fibers in the laminar (La), magnocellular (MCC) and angular (Ang) nuclei (related auditory nuclei). Scale bar: 800 µm.

No label was found in the dorsal LVN (Fig. 5), but many fibers were identified in the ventral portion, specifically, at their rostral portion (Fig. 4b). Just a few labels were observed at the caudal LVN (Fig. 5). Although some of these were thin and long, apparently crossing lateral to medial to probably reach the medial vestibular nucleus, many large ones were disposed at the central part, the same place where several varicose basket-like terminals were observed (gray arrow head in Fig. 2b).

### Medial Vestibular Nucleus (MVN)

The rostral MVN hold limits with the SVN and brachium conjunctive, presenting very few thin-like with and without varicose (Fig. 4a).

Moving to the caudal to initiate the intermediate portion, just into the appearance of the LVN, the number dramatically increases and many varicose can be observed in the tiny fibers as well many terminal-like arborizations can now be observed (Figs. 4b and 5). It was noticed that HSCC fibers seemed to be concentrated in the medial instead of the lateral part just below the fourth ventricle (Fig. 4b).

### Descendent Vestibular Nucleus (DVN)

Many BDA-containing fibers were detected all over the DVN (Fig. 5). The rostral and caudal DVN portions presented all three fiber-types, and they seemed to be enriched in the central area of the nucleus, rising in number as they achieve to the caudal portion. Both, large and thin fibers were found within the entire nucleus but the terminal-like structures seem to avoid the lateral limits of the DVN. These basket terminals are full of intense labeled varicose and appear to surround an unlabeled cell (gray arrow head Fig. 2b).

### Occurrence and distribution of the immunoreactivity to AMPA-type subunits

**Figure 6:**
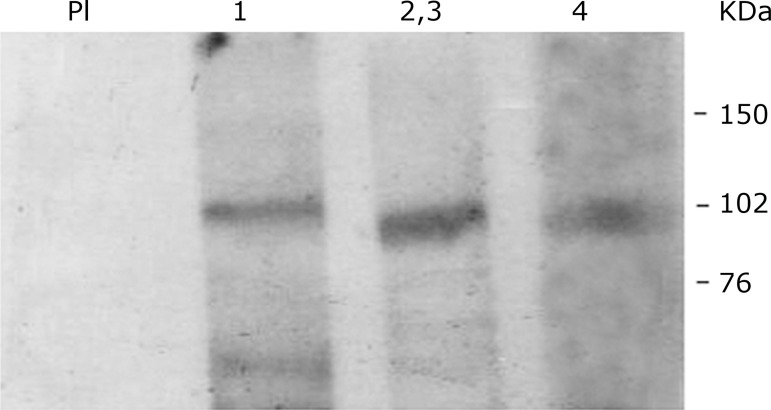
Test representative of at least four independent tests performed with the brainstem of adult pigeons. Thirty milligrams of cellular protein from the total extract were used in SDS PAGE. After being transferred to a nitrocellulose membrane, the extracts were incubated with anti GluR1 (1), anti GluR2/3 (2,3), anti GluR4 (4) and preimmune serum (PI) 1:500. Development was performed using ECL western blotting detection reagent (GE Healthcare). Full-Range Rainbow Molecular Weight Markers (RPN 800, Sigma) was used and the range of interest pointed to the right. The bands for each receptor subunit were obtained as expected: 105–107 κDa for GluR1, 98–100 κDa for GluR2/3 and 100 κDa for GluR4.

The immunoblotting assay shows a good reactivity for all three immune-markers studied further, indicating the constitutive expression of these AMPA receptors in the hindbrain region of the adult pigeons (Fig. 6).

**Figure 7:**
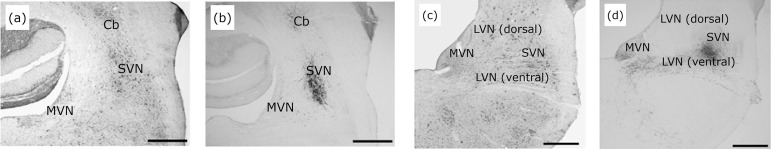
Fiber distribution pattern projecting from the horizontal semicircular canal (HSCC), marked with anterograde PDA, in the rostral portion of the VNC. Images in **(a)** and **(c)** show Giemsa-stained cross-sections: superior vestibular nucleus (SVN), lateral vestibular nucleus (LVN), medial vestibular nucleus (MVN) and cerebellum (Cb). In the SVN, a greater density of long and fine fibers, containing numerous varicosities and few terminal branches in the mid-dorsal region of the rostral pole **(b)**. The MVN receives fibers filled with PDA throughout its entire length, with few projections and nonvaricose fibers in the rostral portion **(b)** and with a marked increase in fibers with many varicosities in the intermediate region of the nucleus **(d)**. In the rostral pole of the LVN (ventral) there are extensive projection fibers mainly concentrated in the ventrolateral nucleus with no projections in the dorsal nucleus **(d)**. Scale bar: 500 μm.

**Figure 8:**
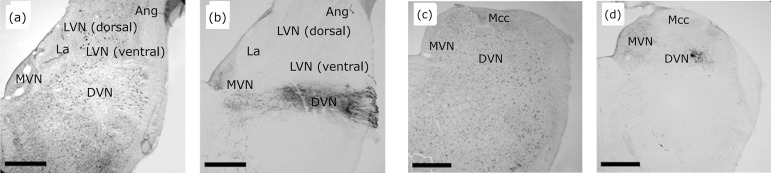
Distribution pattern of horizontal semicircular canal projection fibers (HSCC), stained with PDA, in the caudal VNC. Images **(a)** and **(c)** show Giemsa-stained cross-sections: lateral vestibular nucleus (LVN), medial vestibular nucleus (MVN), descending vestibular nucleus (DVN), angular nucleus (Ang), laminar nucleus (La) and magnocellular nucleus (MCC). Images **(b)** and **(d)** demonstrate the absence of HSCC projections to the Ang, La, MCC, dorsal LVN nuclei and few fibers projecting to the caudal pole of the ventral LVN. Scale bar: 1 mm.

The occurrence of AMPA-type containing neurons coincides with the massive location of the terminal fields of the BDA-filled fibers assuming to be HSCC projecting terminals (Figs. 7 and 8).

Figure 7a shows that the terminal bottom of HSCC projections, filled with BDA surround VNC neurons, and are positively immune-labeled to GluR2/3 (Fig. 7b) and GluR4 (Fig. 8a), but not to GluR1 (Fig. 8b).

## Discussion

### Afferent vestibular inputs to the VNC

Several aspects of the VNC, like cellular morphometric and input-output connection, have been extensively described in numerous species^1^^,^ 2 ^,^ 4 ^,^ 16 ^,^ 28 ^-^ 31, with not so much to add to the neurochemical attributes of these findings. Current data, moreover, authenticate that many of the VNC neurons contain AMPAr subunits with a distinct pattern of occurrence that matches the area in which arriving inputs from the HSCC take place.

Analyzing the VG neurons, we also observed that the presence of bipolar neurons held distinct sizes, all of them fulfilled BDA, occupying the superior dorsal area of the ganglion as predicted by Dickman and Fang^4^ while also in accordance with that reported for primates^32^.

After passing by the restiform body and the rostral part of the tangential nucleus, the BDA filled fibers comprise two paths: 1) run lateral to medial, adjoining the central and caudal portions of the VNC; 2) ascend dorsal to rostral to arrive at the most lateral and superior portion of the VNC.

Even presenting minor differences in innervations pattern and terminal fields, the HSCC projections achieve possible synaptic contacts with neurons belonging to all nuclei of the VNC, except those present in the dorsal LVN. This feature of the double vestibular input appears to be a very conservative archetype already described in primates^2^^,^ 32 ^,^ 33 and avian^1^. However, in the brains of chinchilla^3^ and pigeon^4^ they both report an apparent lack of vestibular primary innervation of LVN dorsal portion.

As long as the fibers reach their target, they grow tiny varicosed branches acquiring a constitution of terminal fields. In the SVN these rich varicose fibers were found preferentially in the central-medial portion while in the LVN they were, in majority, found in the central-dorsal region in the ventral portion since the dorsal LVN seems to be a recipient free zone from peripheral terminals. In the MVN these structures were found in the dorsomedial portion just below the fourth ventricle while the entire DVN, especially in the central part, seems to be tagged, although, decreasing in intensity along their rostral-caudal axis. Again, these broad descriptions come into a conservative element being similar to some species of monkeys^2^^,^ 32, rodents^3^, and marsupials^28^, consenting of previous reports in avian species^1^^,^ 4.

A few discrepancies were observed; for instance, HSCC in chinchillas, in which there is a supposed preference of rostral innervation in the LVN form^3^, and in the squirrel monkey, of which there was described an entire innervation in the MVN besides a caudal preference in the DVN^33^. Although these differences could be easily credited to species variation (probably derived from functional adaptations, and more than expected to have been found), it is important to noticed that these two studies were not dealing with HSCC exclusive projections.

### VNC neurons containing AMPArs as primary target for HSCC afferents

Since the peripheral vestibular inputs are carried out by the neurotransmitter glutamate^20^^,^ 34 ^-^ 37, the glutamate receptors play a key role in such a process. We have mapped the AMPA-type immuno-label distribution in the vestibular hindbrain area of chicks^16^ and found the vast prevalence of the subunit GluR4 and the GluR2/3 expression in components of the VNC in this species, in the same way as in mammals^17^^,^ 36 ^,^ 38 ^,^ 39.

**Figure 9:**
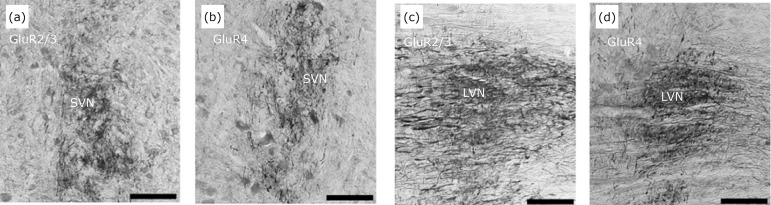
Area of predominance of endings of horizontal semicircular canal projections (HSCC) in regions of cells immunoreactive to GluR2/3 and GluR4 subunits in the VNC. The projection fibers reach the mid-dorsal portion of the rostral pole of the superior vestibular nucleus (SVN), where the cells are immunoreactive to GluR2/3 **(a)** and GluR4 **(b)**. Projecting fibers at the rostral pole of the lateral vestibular nucleus (LVN) ending in cells immunoreactive to the GluR2/3 **(c)** and GluR4 **(d)** subunits. Scale bar: 80 μm.

GluR2/3 and GluR4-expressing neurons are uniformly distributed over all lengths in SVN, and the central part of these nuclei is the place in which we found a preponderance of BDA-filled terminal fibers (Fig. 9). In addition, Popper *et al*.^17^ described that both markers were preferentially found in the central area of the rostral pole with no distinction for chinchillas.

**Figure 10:**
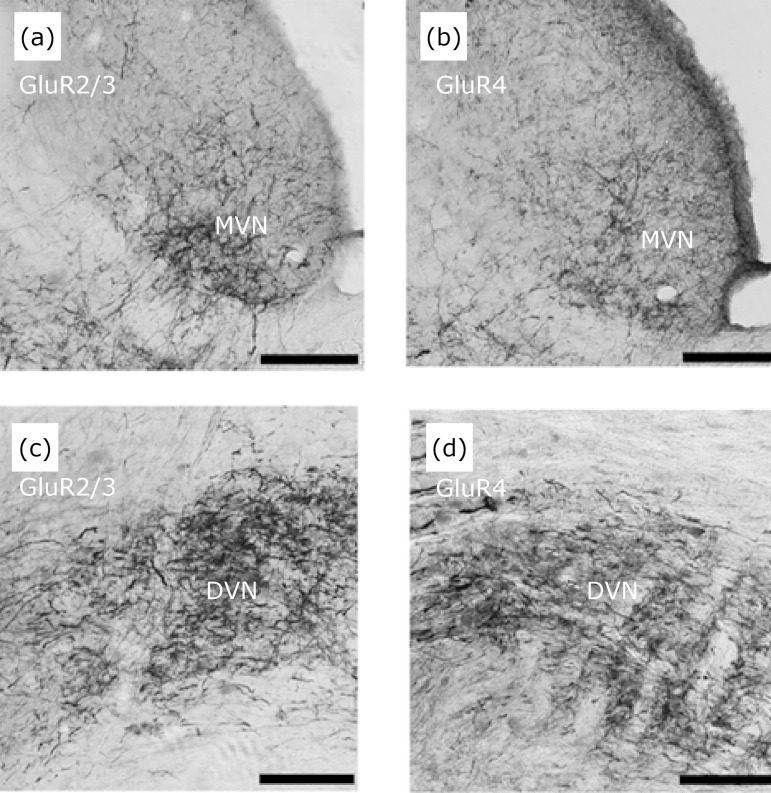
Area of predominance of terminations of horizontal semicircular canal projections (HSCC) in regions of cells immunoreactive to GluR2/3 and GluR4 (1:250) subunits of the VNC. HSCC projection fibers ending in areas containing neurons immunoreactive to GluR2/3 and GluR4 are observed in the intermediate and caudal portions of the medial vestibular nucleus (MVN) (a and b) and rostral portion of the descending vestibular nucleus (DVN) (c and d). Scale bar: 80 µm.

**Figure 11:**
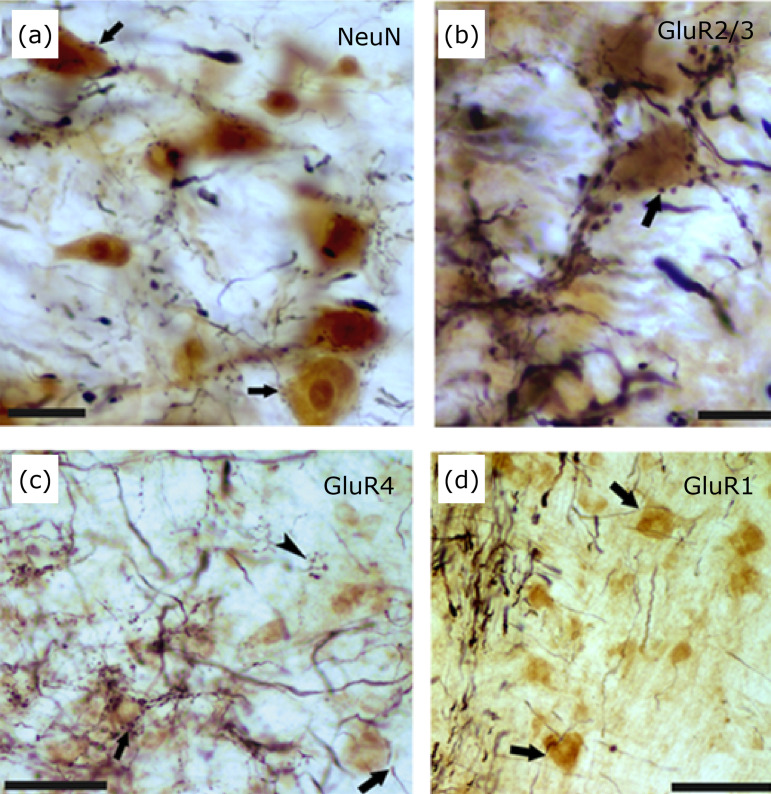
Photomicrographs of cross-sections of the lateral vestibular nucleus (LVN) showing fibers that terminate in cells immunoreactive to AMPA glutamatergic receptors. Cells immunolabeled with NeuN **(a)** and the subunits GluR2/3 **(b)** and GluR4 **(c)** appear surrounded by stained fibers containing BDA in opposition to buttons (*arrows*), it was also observed in the presence of these cells, unmarked terminals (*head of arrow*). In image **(d)**, no termination is observed in GluR1-immunoreactive cells (*arrow*). Scale bars: A and B: 10 µm; C: 50 µm; D: 30 µm.

In the LVN, MVN and DVN (Figs. 10 and 11) we observed a homogeneous distribution of neurons enriched in GluR2/3 and GluR4^16^ at the same terminal field-like fibers filled with BDA, again, in concordance with that reported by Popper *et al*.^17^ in chinchilla and by Chen *et al*.^19^ in rat brain.

### Functional considerations

The efficiency of the information carried out by the HSCC inputs in controlling head movements with ocular adjustments and their influence in postural tonus depends not just in the final destination of these fibers but in the neurochemical nature of these connections as well.

GluR2/3 and GluR4 subunits of AMPA receptors were found in all targeting regions of the HSCC in all pigeon vestibular nuclei suggesting its involvement in excitatory postsynaptic transmission in vestibular neurons, similar to findings in other species^16^^,^ 18 ^,^ 40 ^,^ 41. While recognizing the mediating role of the VNC in posture adjustments and balance, each individual nucleus plays an important role in different controls at this function.

The SVN is considered the most convey center for the integration of ocular reflexes mediated by semicircular canals, thus, being the cranial motor nuclei one of the SVN efferent targets^33^. Therefore, by the oculomotor, trochlear, and abducent somatic efferent the extraocular muscles move the eye in response to vestibular stimulus^28^. As the pattern of the HSCC terminal fiber in the SVN is the central, and equally noted the prevalence of magnocellular neurons in this portion, it is possible to suggest that those neurons might be implicated with the mediation of these adjusts, corroborating earlier predictions by Popper *et al*.^17^.

The LVN participates in modulating postural adjustments of the neurons of the spinal cord through vestibulospinal lateral tract, consisting of projections of their dorsal and ventral portions^4^^,^ 31 ^,^ 42. According to our present data and from others^1^^,^ 3 ^,^ 28, only the ventral portion of the LVN receives HSCC projections. Neurons and their design are predominantly in the lumbosacral region of the spinal cord^2^^,^ 15 ^,^ 43, suggesting that the vestibular input sensors may be involved in adjusting the dynamic tone by spinal cord cells.

By the medial vestibulospinal tract, the MVN (with a small bunch of DVN fibers) influences the postural control and the horizontal vestibulocochlear reflex by sending projections to the cervical and thoracic regions of the spinal cord^42^^,^ 43. Electrophysiological studies demonstrated that such a reflex is almost strictly dependent of the displacement of the cupula of the HSCC during angular acceleration of the head in attention to the vertical axis^37^^,^ 44 ^,^ 45.

Different subunits of the vestibular nuclei glutamatergic receptors are involved in synaptic formation during the development and in synaptic plasticity both for in normal and pathological conditions^38^^,^ 46 ^,^ 47. The functional properties of the AMPA receptors are primarily determined by GluR1 and GluR4 corresponding to the high influx of Ca^++^ in the vestibular immature neurons, coinciding with the absence of GluR2 for first day postnatal^38^^,^ 47. In late periods of postnatal development, the progressive reduction of the permeability to Ca^++^ channels AMPA is due to increase expression of the subunit GluR2 in the neurons vestibular nuclei^18^^,^ 48 and decrease expression of the GluR1 subunit^38^.

## Conclusions

Our data show that three different types of BDA filled fibers, from HSCC, spread for all CNV nuclei, except for the dorsal portion of lateral vestibular nucleus. The presence of terminals fields in CNV neurons was evidenced after GluR2/3, GluR4 and NeuN immunoreactivity.

Projections of HSCC for CNV are the main output signal to the spinal cord, reinforcing the information that the vestibular system has strong relation with the fast responses necessary for postural control. Moreover, its phylogenetic organization apparently conservation, also seems to be a fundamental characteristic for vertebrates.
